# Supplementary material for: WordCommentsAnalyzer: A windows software tool for qualitative research
Source: F1000Res. 2018 Sep 4;7:536. Originally published 2018 May 3. [Version 2] doi: 10.12688/f1000research.14819.2 (PMC6137411; doi:10.12688/f1000research.14819.2)
Supplement: Supplementary file 3 [file f1000research-7-17614-s0002.tgz › b2a79496-4ada-47c4-9682-a99ce867836d.zip!/Eight Iranian Health Journals/Archives of Rehabilitation_10.docx]

© Archives of Rehabilitation (RJ). University of Social Welfare and Rehabilitation Sciences, Koodakyar Alley, Daneshjoo Blv., Evin, Tehran, Iran. This work is licensed under [CC BY-NC 4.0](https://creativecommons.org/licenses/by-nc/4.0/).

Volume & Issue: دوره 16 - شماره 4

Title: نامه به سردبیر: مراقب همراه: دستاوردی نوین در هندلینگ مادری کودکان دارای فلج مغزی

http://rehabilitationj.uswr.ac.ir/article-1-1725-fa.html

آموزش از طریق تلفن همراه و برنامه های کاربردی سلامت همراه، یکی از عناصر اصلی در آموزش الکترونیک  می باشند.  برای اولین بار در ایران، نرم افزار مراقب همراه، ویژه هندلینگ مادری از کودک دارای فلج مغزی با همکاری متخصصان کاردرمانی و فناوری سلامت، طراحی و پیاده سازی شد. برای طراحی نرم افزار هندلینگ مادری از کودک فلج مغزی، از نسخه 13 نرم افزار InteliJIDe در محیط اندروید استفاده شد. این نرم افزار شامل بخش های آشنایی با کودک دارای فلج مغزی، اصول تکامل حرکتی، تغذیه، لباس پوشیدن، سرویس بهداشتی، حمام کردن، خواب، حمل و جابجایی، بازی، داروهای ضد تشنج و درباره ما می باشدکه از طریق بلوتوث و بصورت رایگان قابلیت نصب بر روی تلفن های همراه و تبلت اندروید را دارد. این نرم افزار گامی مهم در دسترسی آسان، سریع و رایگان خانواده ها به اطلاعات مراقبت های ویژه روزمره از کودکان دارای فلج مغزی است.  امید است با استفاده از آن، بخشی از مشکلات این خانواده ها مرتفع گردد.

Title: شیوع ناهنجاریهای سر و اندام فوقانی در دانشآموزان با نیازهای ویژه

http://rehabilitationj.uswr.ac.ir/article-1-1639-fa.html

هدف: ناهنجاری‌های عضلانی-اسکلتی از ‌گسترده‌ترین مشکلات دوران کودکی محسوب می‌شود. هدف از پژوهش حاضر بررسی شیوع ناهنجاری‌های اندام فوقانی در دانش‌آموزان با نیازهای ویژه است. روش بررسی: روش بررسی مقاله حاضر، روش مقطعی-توصیفی با نمونه‌گیری طبقه‌ای نسبتی است. نمونه‌ها برحسب ده درصد از دانش‌آموزان سال تحصیلی 92-1391 از میان سه اختلال آسیب بینایی، آسیب شنوایی و کم‌توانی ذهنی انتخاب شدند که در شهر تهران 1194 نفر، در شهرستان‌های استان تهران 1526 نفر و در استان البرز 559 نفر بودند. درمجموع، 3279 نفر شرکت‌کننده در مطالعه حضور داشتند. برای جمع‌آوری داده‌ها از پرسشنامه دموگرافیک و شاقول استفاده شد و نتایج با استفاده از آمار توصیفی گزارش گردید. یافته ها: نتایج پژوهش نشان داد که «افتادگی شانه» در دخترانِ با آسیب بینایی در استان البرز با % 100 بیشترین میزان و در پسرانِ با آسیب بینایی شهر تهران کمترین میزان شیوع را با % ۳۴٫۹  داشت. در «ناهنجاری‌های سر»، در انحراف سر به طرفین با%۷۶٫۶ دخترانِ با آسیب بینایی شهر تهران % ۷۶٫۶ و در «ناهنجاری سربه‌جلو» پسرانِ با آسیب بینایی شهرستان‌های تهران با % ۳۸٫۳، بالاترین درصد شیوع را در مقایسه با دیگران داشتند نتیجه گیری: ناهنجاری‌های اندام فوقانی در دانش‌آموزانِ با نیازهای ویژه اعم از کم‌توان ذهنی، آسیب بینایی و شنوایی بالا بوده است و تشخیص و درمان به موقع این ناهنجاری‌ها ضروری است.

Title: اثر تمرینات گروهی و تنظیم بریس میلواکی در فواصل کوتاه‌تر بر شدت انحنا و کیفیت زندگی بیماران مبتلا به اسکولیوز ایدیوپاتیک

http://rehabilitationj.uswr.ac.ir/article-1-1640-fa.html

هدف:بریس بهمراه ورزش مؤثرترین پروتکل درمانی اسکولیوز ایدیوپاتیک برای بیماران با زوایای اسکولیوزی 45-25 درجه می‌باشد.بهرحال جنبه‌های روانشناختی درمان اسکولیوز که کیفیت‌زندگی را نیز تحت تاثیر قرار خواهد داد، و نامشخص بودن زمان دقیق تنظیم نیروی پدهای بریس میلواکی،هدف این مطالعه چندوجهی را بر پایه بررسی اثر انجام این ورزش‌ها بصورت گروهی همراه با تنظیم بریس میلواکی در فواصل کوتاهتر در مقایسه با روش مرسوم قرار داده است. روش بررسی:سی و چهار بیمار مبتلا به اسکولیوز ایدیوپاتیک با زوایای اسکولیوزی 50-15 درجه در این مطالعه وارد شدند و در دو گروه آزمون و کنترل قرار گرفتند.افراد دو گروه در یازده هفته برنامه درمانی، متفاوت بین دو گروه،شرکت نمودند.کیفیت‌زندگی افراد هر دو گروه،قبل و بعد از مداخله با تکمیل پرسشنامه SRS-22،و همچنین زوایای اسکولیوزی آنها قبل و بعد از مداخله بر اساس عکس‌های رادیوگرافی اولیه و ثانویه مورد ارزیابی و مقایسه قرار گرفت.جامعه آماری،بیماران مراجعه کننده به مرکز جامع توانبخشی هلال احمر و نمونه‌گیری به صورت غیرتصادفی صورت گرفت. یافته‌ها:شدت انحنای اسکولیوزی گروه آزمون در مقایسه با گروه کنترل،بعد از مداخله بطور معناداری کاهش یافته‌است(p=0.04).در حوزه‌ی رضایت‌از‌درمان نیز بین دو گروه بعد از مداخله اختلاف معناداری مشاهده شد(p=0.04).همچنین در خصوص کیفیت‌زندگی بیماران با زوایای کاب کمتر از 30 درجه در مقایسه با بیماران با زوایای کاب بالاتر از 31 درجه،در حوزه‌های خویشتن‌بینی(p=0.00)،رضایت‌از‌درمان(p=0.03) و نمره‌کلی کیفیت-زندگی(p=0.00)،تفاوت معناداری مشاهده گشت. نتیجه‌گیری:تنظیم بریس در فواصل کوتاهتر همراه با انجام ورزشها به صورت گروهی،در طی یازده هفته موجب افزایش رضایت از درمان و کاهش زاویه انحنای اسکولیوزی بیماران مورد مطالعه شده‌است.

Title: سنجش تکرارپذیری نسخه فارسی پرسشنامه «ابزار بی ثباتی مچ پای کامبرلند» در ورزشکاران ایرانی مبتلا به پیچخوردگی خارجی مچ پا

http://rehabilitationj.uswr.ac.ir/article-1-1641-fa.html

روش بررسی: این پژوهش، پژوهشی پیمایشی از نوع هنجاریابی آزمون است. در این مطالعه، پس از ترجمه پیشرو و پسرو «پرسشنامه بی‌ثابتی مچ پای کامبرلند»، 46 ورزشکار به روش نمونه‌گیری در دسترس از خانه تکواندوی تهران و دانشکده تربیت‌بدنی دانشگاه تهران انتخاب و شرکت داده شدند. پرسشنامه به افراد شرکت‌کننده‌ای که براساس تشخیص پزشک، حداقل یک‌بار سابقه پیچ‌خوردگی خارجی مچ پا را داشتند، داده شد. در مرحله دوم (یک هفته بعد‌) پرسشنامه دومرتبه بین شرکت‌کنندگان توزیع شد تا پایایی بین دو آزمون سنجیده شود. پس از جمع‌آوری داده‌ها، تکرارپذیری آزمون-بازآزمون نسخه فارسی پرسشنامه با محاسبه ضریب همبستگی درون‌گروهی، خطای معیار اندازه‌گیری و کمترین تغییرات قابل قبول، بررسی و ضریب آلفای کرونباخ به‌منظور ارزیابی همخوانی درونی سؤال‌های پرسشنامه با استفاده از نسخه 20 نرم ‌افزار SPSS نسخه 20 محاسبه شد. یافته ها: ضریب آلفای کرونباخ 64/0 است که به سطح قابل قبول برای همخوانی درونی در سطح آیتم‌ها (95/0–7/0) بسیار نزدیک است. تحلیل عامل نشان داد با حداکثر 72%  پوشش واریانس می‌توان سؤالات پرسشنامه بی‌ثباتی مچ پای کامبرلند را در 4 دسته قرار داد. برای نمره کل CAIT، ضریب همبستگی ICC در آزمون-بازآزمون 95/0 (001/0>P) محاسبه شد که نشان‌دهنده تکرارپذیری عالی نسـخه فارسی پرسشنامه است. خطای معیار اندازه‌گیری (SEM) عدد 1 و کمترین تغییرات قابل قبول (SDC) با %95 اطمینان 76/2 به‌دست آمد. نتیجه گیری: نتایج پژوهش حاضر نشان می‌دهد که نسخه فارسی پرسشنامه بی‌ثباتی مچ پای کامبرلند، می‌تواند به‌عنوان ابزاری پایا برای تشخیص بی‌ثباتی و سنجش تغییرات ناشی از مداخلات درمانی در ورزشکاران مبتلا به بی‌ثباتی فانکشنال مچ پا مورد استفاده قرار گیرد.

Title: اثر تمرین اکستنشن ایزوکینتیک زانو با حداکثر چرخش خارجی تیبیا بر آمپلیتیود عضلات وستوس در مبتلایان به سندروم درد پاتلوفمورال

http://rehabilitationj.uswr.ac.ir/article-1-1623-fa.html

هدف: سندروم درد پاتلوفمورال از رایج‌ترین اختلالات مزمن زانو به‌ویژه در میان زنان است که ارتباط تنگاتنگی با ایمبالانس نیروی عضلات وستوس داخلی و خارجی دارد. هدف تحقیق حاضر، بررسی اثر تمرین اکستنشن ایزوکینتیک زانو در حداکثر چرخش خارجی تیبیا بر آمپلی‌تیود عضلات وستوس داخلی و خارجی در مبتلایان به این سندروم بود. روش بررسی: در این تحقیق 36 زن مبتلا به سندروم درد پاتلوفمورال به‌طور داوطلبانه شرکت کردند و در یکی از 3 گروه تمرین اکستنشن ایزوکینتیک مبتنی‌برتقویت انتخابی عضله پهن داخلی مایل، تمرین تقویت عمومی عضله چهارسر و کنترل به‌طور تصادفی قرار گرفتند. این تمرینات به مدت 8 هفته اجرا گردید. آمپلی‌تیود عضلات پهن داخلی مایل و پهن خارجی و نیز نسبت فعالیت پهن داخلی مایل به پهن خارجی قبل و پس از مداخلات تمرینی با استفاده از یک سیستم الکترومایوگرافی 8 کاناله ثبت و محاسبه گردید. تحلیل داده‌های حاصل با استفاده از تحلیل واریانس با اندازه‌گیری مکرر صورت پذیرفت. یافته ها: در ابتدای مطالعه آمپلی‌تیود پهن داخلی مایل، در هر سه گروه کمتر از پهن خارجی (01/0&ge;P) بود، اما پس از مداخلات در گروه تقویت انتخابی بیشتر از پهن خارجی (01/0=P) و در گروه عمومی (001/0=P) و کنترل (036/0=P) کمتر از پهن خارجی به‌دست آمد. قبل از مداخلات، بین گروه‌های مختلف تفاوتی وجود نداشت(05/0<P)، ولی پس از مداخلات، آمپلی‌تیود پهن داخلی مایل و نسبت فعالیت پهن داخلی مایل به پهن خارجی در گروه انتخابی بیشتر از گروه عمومی و کنترل (001/0&ge;P) و آمپلی‌تیود پهن خارجی در گروه عمومی بیشتر از گروه انتخابی و کنترل بود (01/0&ge;P). نتیجه گیری: براساس یافته‌های پژوهش حاضر، می‌توان تمرین اکستنشن ایزوکینتیک در حداکثر چرخش خارجی تیبیا و در نزدیکی فول‌اکستنشن زانو را به‌عنوان تمرینی مناسب برای بهبود نسبت فعالیت الکتریکی پهن داخلی مایل به پهن خارجی و کاهش ایمبالانس بین آمپلی‌تیود عضلات پهن داخلی و خارجی برای مبتلایان به این سندروم پیشنهاد نمود.

Title: مقایسه الگوی فعالیت الکترومایوگرافی عضلات دومفصلی زانو بین جوانان و سالمندان در سرعتهای مختلف راه رفتن

http://rehabilitationj.uswr.ac.ir/article-1-1741-fa.html

هدف: تحلیل راه‌رفتن در‌ سال‌های اخیر، بسیار مورد توجه قرار گرفته است. شاخص‌هایی از قبیل سرعت، سن و جنسیت همگی روی معیارهای راه‌رفتن تأثیر دارند. هدف این پژوهش، مقایسه الگوی فعالیت الکترومایوگرافی عضلات دومفصلی زانو بین جوانان و سالمندان در سرعت‌های مختلف راه‌رفتن است. روش بررسی: روش تحقیق حاضر، روش علّی-مقایسه‌ای است که در آن، تعداد 15 مرد سالمند و 15 مرد جوان سالم به‌صورت موارد در دسترس انتخاب گردید. فعالیت الکتریکی عضلات راست­رانی، دوسررانی، نیم وتری و دوقلو، در حین راه‌رفتن با سرعت خودانتخابی (100%)، کُند (80%) و سریع (120%) در یک مسیر 10 متری با استفاده از یک سیستم الکترومایوگرافی، ثبت گردید. فعالیت الکتریکی عضلات به‌صورت ریشه میانگین مجذورات (RMS) با آزمون‌های تحلیل واریانس با اندازه‌گیری مکرر و توکی در نرم‌افزار SPSS نسخه 18 مقایسه شد. یافته ها: مطابق نتایج، RMS عضلات راست‌رانی در مرحله میانه ایستایش (01/0>P) و دوقلو در مرحله انتقال وزن (02/0=P) در سالمندان در همه سرعت‌های راه‌رفتن بیشتر از جوانان بود و در هر دو گروه سنی با افزایش سرعت، افزایش یافت (01/0>P). RMS   عضله دوسررانی سالمندان در مرحله انتهای نوسان  در سرعت 80% ،کمتر از جوانان بود (01/0=P) و با افزایش سرعت راه‌رفتن، افزایش یافت (01/0=P). RMS عضله سمی‌تندینوس سالمندان در مراحل انتقال وزن و میانه ایستایش در سرعت 120% بیشتر از جوانان بود (01/0>P) و در هر دو گروه سنی در مرحله نوسان  با افزایش سرعت افزایش یافت (05/0>P). نتیجه گیری: براساس نتایج این تحقیق، سالمندان هم‌انقباضی عضلانی بیشتری در اطراف زانو در سرعت بالا و در مرحله میانه ایستایش دارند. این تغییرات وابسته به سن در فعالیت عضلانی، سفتی و پایداری مفصل را طی حمایت یک‌طرفه افزایش می‌دهد و احتمالاً در کاهش توان مرحله «پیشروی» در راه‌رفتن‌های سریع‌تر دخیل باشد.

Title: بررسی روایی و پایایی نسخه فارسی پرسشنامه «کیفیت زندگی افراد ناتوان سازمان بهداشت جهانی» (WHOQOL-DIS) در افراد سالمند ناتوان

http://rehabilitationj.uswr.ac.ir/article-1-1708-fa.html

هدف: تعیین ویژگی‌های روان‌سنجی نسخه فارسی پرسشنامه «کیفیت زندگی افراد ناتوان سازمان بهداشت جهانی» در سالمندان ایرانی مبتلا به ناتوانی، هدف اصلی این پژوهش است. روش بررسی: در مقاله حاضر، با استفاده از روش روان‌سنجی کلاسیک به بررسی روایی و پایایی «پرسشنامه سنجش ناتوانی سازمان بهداشت جهانی» در سالمندان مبتلا به ناتوانی پرداخته شد. در بررسی روایی محتوایی، از دو روش «لاوشه» و «والتس و بازل» استفاده شد. طی نمونه‌گیری دردسترس، 205 سالمندی که نمره «پرسشنامه سنجش ناتوانی سازمان بهداشت جهانی» آنها از 4 بیشتر شده بود، به‌منظور بررسی روایی سازه از نوع همگرا با پرسشنامه SF-12 و روایی ملاکی از نوع مقایسه گروه‌های شناخته‌شده (سن، جنس، گزارش فرد از وضعیت سلامتی و ناتوانی خود، تأثیر ناتوانی) پرسشنامه را تکمیل کردند. برای تعیین پایایی درونی و آزمون-بازآزمون به‌ترتیب از آلفای کرونباخ و ضریب همبستگی درون‌رده‌ای (ICC) و برای تحلیل نتایج از نرم افزار SPSS نسخه 20 استفاده شد. یافته‌ها: در بررسی روایی محتوایی طبق روش لاوشه و والتس و بازل برای همه آیتم‌ها نمره قابل قبولی به‌دست آمد. بین پرسشنامه WHOQOL-DIS و SF-12 غیر از بُعد اجتماعی (13/0=P) همبستگی قابل قبولی (001/0>P) وجود داشت. ضریب همبستگی درون‌رده‌ای (ICC) برای نمره ابعاد پرسشنامه و نمره کل بیشتر از 70/0 بود. آلفای کرونباخ به‌منظور بررسی همخوانی درونی پرسشنامه برای کلیه ابعاد به‌جز بُعد اجتماعی بالای 70/0 بود. مقایسه بین گروه‌های شناخته‌شده، حاکی از وجود تمایز بین جنسیت، وضعیت ناتوانی و سلامتی در بین سالمندان است (001/0>P). نتیجه‌گیری:  براساس یافته‌های این پژوهش، به‌نظر می‌رسد که پرسشنامه «کیفیت زندگی افراد ناتوان سازمان بهداشت جهانی» از روایی ظاهری، محتوایی، سازه، ملاکی و پایایی قابل قبولی در سالمندان مبتلا به ناتوانی برخوردار باشد؛ بنابراین، می‌توان از این پرسشنامه در تحقیقات مربوط به کیفیت زندگی سالمندان مبتلا به ناتوانی استفاده نمود.

Title: ثبات عملکردی اندام فوقانی در زنان والیبالیست با و بدون ناپایداری قدامی شانه با تأکید بر دست برتر

http://rehabilitationj.uswr.ac.ir/article-1-1774-fa.html

هدف: هدف از تحقیق حاضر مقایسه ثبات عملکردی شانه دست برتر و غیربرتر در دو گروه سالم و دارای ناپایداری قدامی شانه توسط آزمون YBT-UQ بود. روش بررسی: در این مطالعه توصیفی ، 28 والیبالیست زن دانشجو 20 تا 25 سال در دو گروه 14 نفره با و بدون ناپایداری قدامی شانه به صورت هدفمند انتخاب شدند. آزمون ثبات عملکردی اندام فوقانی (YBT-UQ) از سمت برتر و غیربرتر افراد گرفته شد. پس از محرز شدن نرمال بودن توزیع داده‌ها توسط آزمون کولموگروف-اسمیرنوف، از آزمون تی زوجی برای مقایسه دست برتر و غیربرتر و آزمون تی مستقل برای مقایسه نتایج دو گروه استفاده شد. یافته‌ها: بیشترین نتایج نمره ترکیبی آزمون به ترتیب در اندام غیربرتر افراد سالم، اندام برتر افراد سالم، اندام غیربرتر افراد آسیب دیده و اندام برتر (آسیب‌دیده) افراد آسیب دیده مشاهده شد. در گروه سالم تفاوت معنی داری بین ثبات عملکردی اندام برتر و غیربرتر مشاهده نشد (p=0.144) در صورتیکه ثبات اندام غیربرتر گروه آسیب‌دیده به طور معنی‌داری بهتر از ثبات اندام برتر یا به عبارتی اندام آسیب دیده بود (p=0.001). در تمامی جهات ثبات اندام آسیب دیده نسبت به ثبات اندام برتر افراد سالم کاهش معنی داری داشت در حالیکه جز در جهت فوقانی-جانبی سمت غیربرتر دو گروه با یکدیگر تفاوت معنی داری نداشتند. نتیجه‌گیری: بر اساس یافته‌های پژوهش حاضر ثبات عملکردی در شانه آسیب‌دیده دانشجویان والیبالیست‌ دارای ناپایداری شانه نسبت به شانه سالم آن‌ها و شانه برتر افراد سالم کمتر بوده بنابراین توصیه می‌شود تمرینات ثبات‌دهنده بخصوص در زنجیره حرکتی بسته در برنامه تمرینی این افراد قرار گیرد.

Title: بررسی روایی صوری و همگرای نسخه فارسی چکلیست ارزیابی سریع تنش اداری روسا

http://rehabilitationj.uswr.ac.ir/article-1-1743-fa.html

هدف: هدف از مطالعه مذکور ترجمه، انطباق فرهنگی و بررسی روایی صوری و همگرای چک‌لیست ارزیابی سریع تنش اداری روسا به زبان فارسی است. روش بررسی: مطالعه توصیفی حاضر از نوع پیمایشی است که مطابق با روش IQOLA انجام شد. روش نمونه‌گیری به‌صورت در دسترس بود که نسخه ترجمه‌‌شده روی 100 نفر از کارکنان اداری مورد ارزیابی قرار گرفت. در این مطالعه به‌منظور بررسی روایی همگرا از چک‌لیست رولا و برای بررسی همبستگی بخش‌های این دو چک‌لیست از ضریب همبستگی پیرسون استفاده شد. روایی صوری چک‌لیست طی دو مرحله، ابتدا با نظرسنجی از 5 فرد متخصص در زمینه فیزیوتراپی، کاردرمانی و ارگونومی و سپس ارزیابی چک‌لیست توسط 9 نفر ارگونومیست صورت گرفت. تحلیل داده نیز با نرم افزاز SPSS  نسخه 19 انجام شد. یافته‌ها: نتایج نشان داد که در مجموع ترجمه عبارت و جملات پرسشنامه آسان و کیفیت ترجمه (شامل وضوح ترجمه، کاربرد زبان مشترک، یکسانی مفهومی و کیفیت کلی ترجمه) عبارات و جملات پرسشنامه مطلوب بود. در ادامه، اعتبار صوری نسخه فارسی پرسشنامه مورد بررسی قرار گرفت و منجر به تغییراتی در مواردی از بخش‌ها و چیدمان ظاهری چک‌لیست گردید. در این بررسی ضریب همبستگی بین نمره کلی چک‌لیست روسا و چک‌لیست رولا معنادار بود (7/0=r و 001/0P<). نتیجه گیری: نسخه فارسی چک لیست روسا برای ارزیابی سریع تنش اداری در بین جوامع ایرانی از روایی لازم برخوردار است.

Title: مقایسه ضخامت عضلات اکستانسور گردنی در زنان مبتلا به گردندرد مزمن غیراختصاصی و افراد سالم با استفاده از اولتراسونوگرافی

http://rehabilitationj.uswr.ac.ir/article-1-1757-fa.html

هدف: گردن درد یکی از شایع ترین اختلالات عضلانی اسکلتی است. یکی از عوارض گردن درد مزمن، تغییر ساختار و عملکرد عضلات گردنی است. با توجه به اینکه اندازه‌گیری ضخامت عضله، معیار مناسبی برای ارزیابی تغییرات ساختاری عضله محسوب می‌شود؛ ازاین‌رو، این مطالعه با هدف بررسی ضخامت عضلات اکستانسور گردنی در افراد مبتلا به گردن‌درد مزمن غیراختصاصی و سالم به‌منظور بررسی تفاوت ساختاری بین دو گروه با استفاده از اولتراسونوگرافی انجام گرفت. روش بررسی: در این مطالعه علی-مقایسه ای، 30 داوطلب زن (15 زن مبتلا به گردن‌درد مزمن غیراختصاصی و 15 زن سالم) شرکت کردند. تصویربرداری اولتراسونوگرافی در سطح مهره چهارم گردنی، از عضلات اکستانسور گردن شامل مالتی‌فیدوس، سمی‌اسپاینالیس سرویسیس، سمی‌اسپاینالیس کپیتیس، اسپلنیوس‌کپیتیس و تراپزیوس فوقانی، در حالت استراحت انجام شد. به منظور مقایسه ضخامت عضلات بین دو گروه  از آزمون t مستقل استفاده شد. تجزیه و تحلیل داده ها با استفاده از نرم افزار spss نسخه 20 انجام گرفت. یافته‌ها: ضخامت عضلات مالتی‌فیدوس و سمی‌اسپاینالیس سرویسیس در گروه بیمار نسبت به گروه سالم کمتر بود (01/0=P ,03/0=P). ضخامت عضلات سمی‌اسپاینالیس کپیتیس، اسپلنیوس‌کپیتیس و تراپزیوس فوقانی بین دو گروه تفاوت معناداری نداشت (05/0<P). نتیجه‌گیری: کاهش ضخامت دیده‌شده در عضلات مالتی‌فیدوس و سمی‌اسپاینالیس سرویسیس در افراد مبتلا به گردن‌درد، بیانگر آتروفی و ضعف اکستانسورهای عمقی گردن است. با توجه به اینکه آتروفی در عضلات سطحی مشاهده نشد؛ بنابراین، به‌نظر می‌رسد درمان این بیماران باید متمرکز بر تمرین‌درمانی عضلات عمقی باشد.

Title: تدوین پروتکل مداخلات به هنگام در تأخیر تکامل گفتار و زبان کودکان خردسال: یک تجربه منحصربه فرد در کشور

http://rehabilitationj.uswr.ac.ir/article-1-1898-fa.html

مقدمه:اختلال گفتار و زبان وقتی در دوران کودکی رخ دهد، پیامد های سنگین آن به زودی در زندگی کودک نمایان خواهد شد به تدریج با عبور از دوره بحرانی رشد زبان، یعنی سه سال اول زندگی، از تاثیر مثبت تحریکات محیطی بر رشد و تکامل گفتار و زبان کاسته می شود.شناسایی زود هنگام کودکان در معرض خطر اختلالات تکاملی گفتار و زبان و یا کودکانی که در مراحل ابتدایی تاخیر تکاملی خود قرار دارند، و فراهم نمودن خدمات مداخله به هنگام و پیشگیری ثانویه از معلولیتهای ناشی از این اختلالات بسیار ضروری است.این مطالعه با هدف تدوین و دستیابی به دستورالعملها (پروتکلها)ی مدون مداخلات زودرس برای اختلالات تکامل گفتار و زبان کودکان ایرانی، طراحی و اجرا شد. مواد و روشها:این مطالعه دو فاز اصلی (مروری و کیفی) داشت.در فاز مروری، ابزار جمع آوری داده ها ، موتورهای جستجو بخصوص آنهایی که محل جمع آوری مطالعات مرور سیستماتیک و یا گایدلاینها می باشند بودند.در فاز کیفی، ابزار و روش جمع آوری داده ها برگزاری بحث های متمرکز گروهی و برگزاری راندهای دلفی مکرر با 20 نفر افراد متخصص و صاحب نظر که به صورت هدفمند انتخاب شدند ، در ساب گروههای 7 گانه بود.  یافته ها: نتایج این پروژه تحقیقی به صورت هفت بسته مداخله (پیوست) به تفکیک حوزه هایاختلالات پیش زبانی،  رشدی زبان،  کم شنوایی،  آوایی-واجی، 5 بلع، 6گفتارفلجی و ناروانی ،  ارائه شده است.  نتیجه گیری:اکثر مطالعات انجام گرفته در خصوص کودکان دچار اختلالات گفتار وزبانی در دنیا مؤید ضرورت وجود برنامه‌های آموزش زبان و تمرینات ادراک شنوایی می‌باشند و بر موثر بودن آنها دلالت دارند ولی غالبا به چگونگی انجام این برنامه‌ها به شکل مبسوط نمی‌پردازند. تا قبل از مطالعه حاضر پروتکل منسجم و نظام مند فارسی، بومی و مبتنی بر شواهد که مورد تایید جمعی از متخصصین ذیربط باشد، جهت مداخلات درمانی و توانبخشی کودکان دچار اختلالات گفتار و زبانی در ایران موجود نبود. به دلیل تفاوتهای زبان شناختی در میان زبانهای مختلف دنیا، و نیز تفاوتهای احتمالی مراحل رشد گفتار و زبان در کودکان مختلف دنیا، امکان استفاده مستقیم از منابع خارجی نیز وجود نداشت و نهایتا نیاز به چنین دستورالعملهای بومی به طور چشمگیری احساس می شد.
